# Center-of-mass velocity during the delivery phase in men's javelin throw is not strongly related to deceleration throughout the cross-step phase

**DOI:** 10.3389/fspor.2026.1824228

**Published:** 2026-05-11

**Authors:** Mizuki Makino, Hiroko Takigawa, Takeo Matsubayashi

**Affiliations:** 1 Department of Sports Sciences, Japan Institute of Sports Sciences, Tokyo, Japan; 2 Faculty of Sports and Health Sciences, Chubu Gakuin University, Gifu, Japan

**Keywords:** competition, horizontal velocity, kinematics, last-cross, run-up

## Abstract

**Introduction:**

In javelin throwing, the whole-body center-of-mass velocity at the last rear foot contact, marking the onset of delivery phase, is a key determinant of performance. However, velocity changes during the preceding cross-step phase remain poorly understood. This study aimed to quantify the dynamics of center-of-mass velocity throughout the cross-step phase, identify factors influencing these dynamics, and examine their relationship with velocity at the onset of delivery phase.

**Methods:**

Eighteen right-handed male javelin throwers competing in a national championship were recorded during competition using a digital camera. The final four steps of the cross-step phase were analyzed, and whole-body center-of-mass velocity was calculated. Correlation analyses and repeated-measures ANOVA were performed to assess relationships among variables and to compare within-individual changes.

**Results:**

Horizontal center-of-mass velocity at the onset of delivery phase was strongly correlated with horizontal velocity at the onset of the cross-step phase (r = 0.890, *p* < 0.001), whereas weak correlation was observed with the overall velocity decrease during the cross-step phase (r = 0.249, *p* = 0.319). Additionally, the final cross step exhibited lower velocity than the preceding steps and was strongly correlated with the total velocity decrease throughout the cross-step phase (r = 0.791, *p* < 0.001).

**Discussion:**

These findings suggest that interindividual differences in velocity at the onset of delivery phase are more strongly related to initial cross-step velocity than to the magnitude of velocity decrease during cross-step phase. Nevertheless, because velocity decrease was pronounced during the second half of the cross-step, further investigation into mechanical factors underlying deceleration at these steps may provide additional insight into approach velocity regulation.

## 1 Introduction

The javelin throw comprises three distinct phases: the run-up, cross-step, and delivery (1). Competition performance is primarily determined by the javelin's initial velocity, release angle, release height, distance from the release position to the foul line, and aerodynamic factors during flight (2). Among these, initial velocity is most strongly associated with throwing performance (3–5), whereas release angle and angle of attack, parameters reflecting aerodynamic effects on javelin during flight, exhibit optimal ranges (6). Consequently, increasing initial velocity represents the most critical factor for maximizing javelin throw performance.

Because javelin velocity increases rapidly during the delivery phase (7), most previous studies have focused on factors influencing initial velocity within this phase. These studies have demonstrated that throwers achieving higher initial velocities exhibit greater whole-body center-of-mass (CoM) velocity (3, 8, 9), longer horizontal distance between the javelin grip and CoM at the last front foot touchdown (10), and higher peak velocities at the shoulder, elbow, and javelin (11). Collectively, these findings indicate that landing the front foot with high CoM velocity is crucial for effectively accelerating the javelin through the subsequent overarm throwing motion and achieving high initial velocity.

Notably, Murakami et al. (4) reported a correlation coefficient of 0.742 between throwing performance and CoM velocity at the last rear foot touchdown. Murakami et al. (9) also demonstrated a strong correlation between CoM velocity at front foot contact and that at rear foot contact (r = 0.925). High CoM velocity is advantageous for increasing whole-body mechanical energy, thereby enhancing energy transfer to the javelin during the throwing motion following the last front foot touchdown. Therefore, the mechanical energy ultimately transferred to the javelin is partly determined by CoM velocity developed during the run-up and cross-step phases.

The cross-step is a movement unique to javelin throwing, in which the torso and throwing arm draw the javelin backward while the legs perform a sprinting motion (12). However, studies specifically examining CoM velocity dynamics during the cross-step phase remain limited. Theoretically, CoM velocity at the last rear foot contact, which marks both the end of the cross-step and the onset of the delivery phase, equals the sum of velocity at the onset of the cross-step and the velocity change during this phase. Yet the relative contributions of these two factors remain unclear. Moreover, excessively high CoM velocity that exceeds an athlete's adaptive capacity may result in poor performance. Given this possibility, athletes may adjust their speed just before the delivery phase. The “last cross” step, the final step of the cross-step phase, typically involves larger step length and flight time than preceding steps, and horizontal velocity may decrease. Quantifying these relationships could inform strategies for achieving high CoM velocity at the onset of delivery phase, a key performance determinant in javelin throwing.

Therefore, this study aimed to quantify the dynamics of CoM velocity during the cross-step phase, identify factors influencing these dynamics, and examine their relationship with velocity at the onset of the delivery phase. We tested three hypotheses: (1) throwers with higher horizontal CoM velocity at the final rear foot contact exhibit higher horizontal velocity at the onset of the cross-step phase and smaller velocity decrease throughout; (2) horizontal velocity during the last cross is significantly lower than during the preceding steps; (3) throwers with greater overall velocity decrease during the cross-step phase show greater velocity decrease specifically during the last cross.

## 2 Materials and methods

### 2.1 Participants

Eighteen male javelin throwers participated in this study (the range of throwing record: 63.59–87.16 m). All participants were right-handed and were classifies as tier 3 (highly, international level) or 4 (elite, international level) according to the athlete classification framework (13). Sample size was determined *a priori* using the G*Power software for correlation analysis. Based on a two-tailed test with an effect size of 0.7, an alpha level of 0.05, and a statistical power of 0.8, the minimum required sample size was calculated to be 11 participants. This study was approved by the ethics review committee of the institution with which the first and corresponding authors are affiliated (approval number: 2024–019).

### 2.2 Data collection and processing

Cross-step movements were recorded during competition using a digital video camera (HC-W870M, Panasonic, Osaka, Japan) positioned on the left side of run-up area in the stadium stands. To minimize perspective distortion, the camera was positioned approximately 50 m from the runway. To reduce edge-related distortion, a 2-m margin was included on both sides of the recorded image. The frame rate was set at 60 Hz with an exposure time of 1/1,000 s. The recording area spanned 4–16 m behind the foul line on the run-up pitch. Prior to competition, a calibration pole was positioned and recorded at four locations within this area (4, 8, 12, and 16 m points behind the foul line at the center of the run-up path).

**Figure 1:**
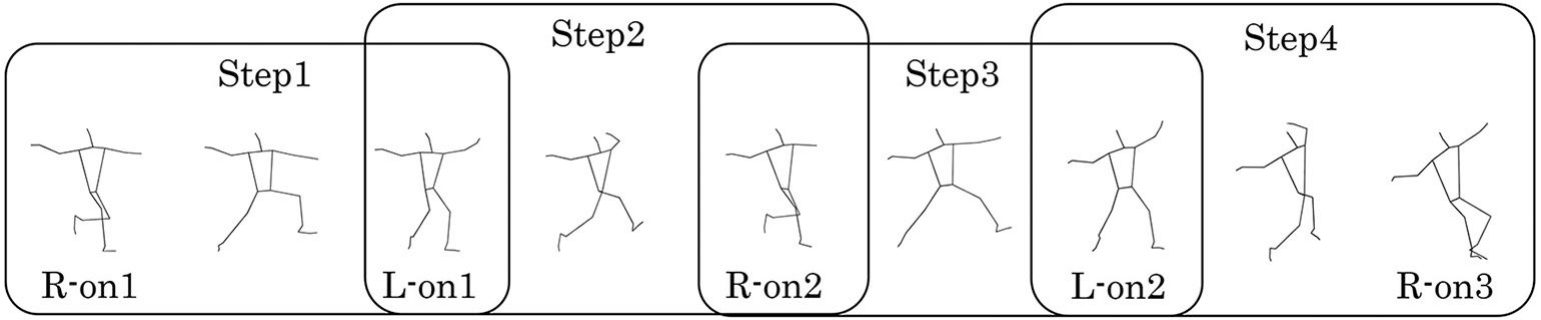
The range of two-dimensional analysis for cross-step phase.

All participants employed a four-step style for their cross-step phase (Figure 1). The best trial of each thrower was selected for analysis. Twenty-three body landmarks were manually digitized on the video images using motion analysis software (Frame-DIAS IV, Q'sfix, Tokyo, Japan). A two-dimensional approach was adopted because the primary variable of interest was horizontal velocity, and the direction of travel was predominantly constrained to a sagittal plane. A two-dimensional coordinate system was established with the horizontal run-up direction as the *X*-axis and the vertical direction as the *Y*-axis. Landmark coordinates were calculated using the two-dimensional direct linear transformation method (14). The calibration error was 0.008 m on *X*-axis and 0.004 m on *Y*-axis. Subsequent processing was performed in MATLAB (version 2019a, MathWorks, Massachusetts, USA). Coordinate data were smoothed using a 2.4–4.2 Hz Butterworth lowpass digital filter, determined by the residual method (15).

The analysis encompassed five key foot-contact events within the cross-step phase: first right foot contact (R-on1) at the onset of the cross-step phase, first left foot contact (L-on1), second right foot contact (R-on2), second left foot contact (L-on2), and final right foot contact (R-on3), which marks both the end of the cross-step phase and the onset of the delivery phase (Figure 1). Four steps were defined: Step1 (R-on1 to L-on1), Step2 (L-on1 to R-on2), Step3 (R-on2 to L-on2), and Step4 (L-on2 to R-on3).

Whole-body CoM position was estimated using body segment inertial parameters for Japanese athletes reported by Ae et al. (16). To reduce noise amplification associated with numerical differentiation of kinematic data sampled at 60 Hz, horizontal CoM velocity during the cross-step phase was quantified using step-averaged velocity rather than instantaneous velocity. For each step, horizontal velocity was determined by calculating the horizontal displacement of the CoM between two consecutive foot-contact events divided by the elapsed time between those events. This measure reflects the net horizontal velocity of the CoM across each step, including both stance and flight phases. Horizontal velocity at the onset of the cross-step phase was defined as the velocity during Step1.

Horizontal velocity at the onset of the delivery phase was defined as the event-specific velocity at the final rear foot contact (R-on3). To enhance robustness against measurement uncertainty associated with numerical differentiation, the velocity at R-on3 was calculated as the mean horizontal CoM velocity from last left foot take-off to R-on3 (mean ± standard deviation: 13.8 ± 2.1 frames). This approach was based on the assumption that horizontal CoM velocity remains nearly constant during the flight phase when aerodynamic effects are neglected. Thus, this procedure provides a locally averaged estimate of instantaneous velocity at the onset of the delivery phase while reducing random measurement error.

The overall change throughout the cross-step phase was defined as Step4 velocity minus Step1 velocity. Step-specific change was calculated as the difference between consecutive step velocities. Negative values indicated net deceleration, whereas positive values indicate net acceleration.

### 2.3 Statistical analysis

The normality of all variables was assessed using the Shapiro–Wilk test. Relationships between variables were evaluated using Pearson's correlation coefficient. Correlation magnitudes were interpreted according to Hopkins et al. (17): trivial (< 0.1), small (≥ 0.1), moderate (≥ 0.3), large (≥ 0.5), very large (≥ 0.7), and extremely large (≥ 0.9). Differences among steps were evaluated using one-way repeated-measures analysis of variance (ANOVA), followed by Bonferroni-adjusted *post hoc* comparisons. Effect sizes were calculated using partial eta-squared ($\eta_{p}^{2}$) for ANOVA and Hedges's g for pairwise comparisons. Effect magnitudes were interpreted according to Cohen (18): small (> 0.01), moderate (> 0.06), and large (> 0.138) for partial eta-squared; small (> 0.2), medium (> 0.5), large (> 0.8) for Hedges's g.

## 3 Results

**Figure 2:**
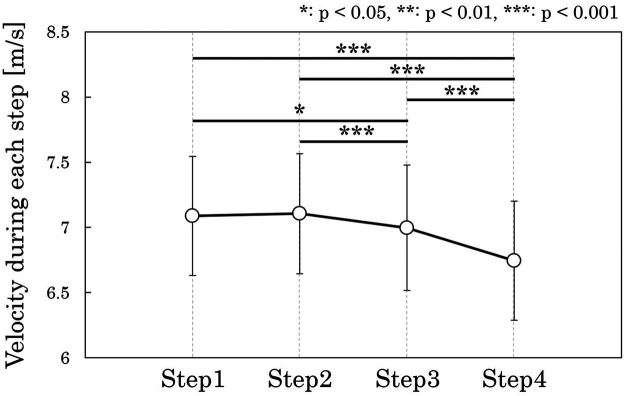
Horizontal center-of-mass (CoM) velocity (mean ± standard deviation) across Step1, 2, 3, and 4 during cross-step phase.

**Figure 3:**
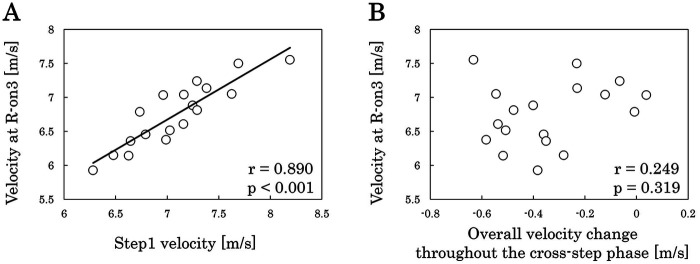
Relationships between horizontal CoM velocity at R-on3 and **(A)** Step1 velocity and **(B)** overall velocity change throughout the cross-step phase.

**Figure 4:**
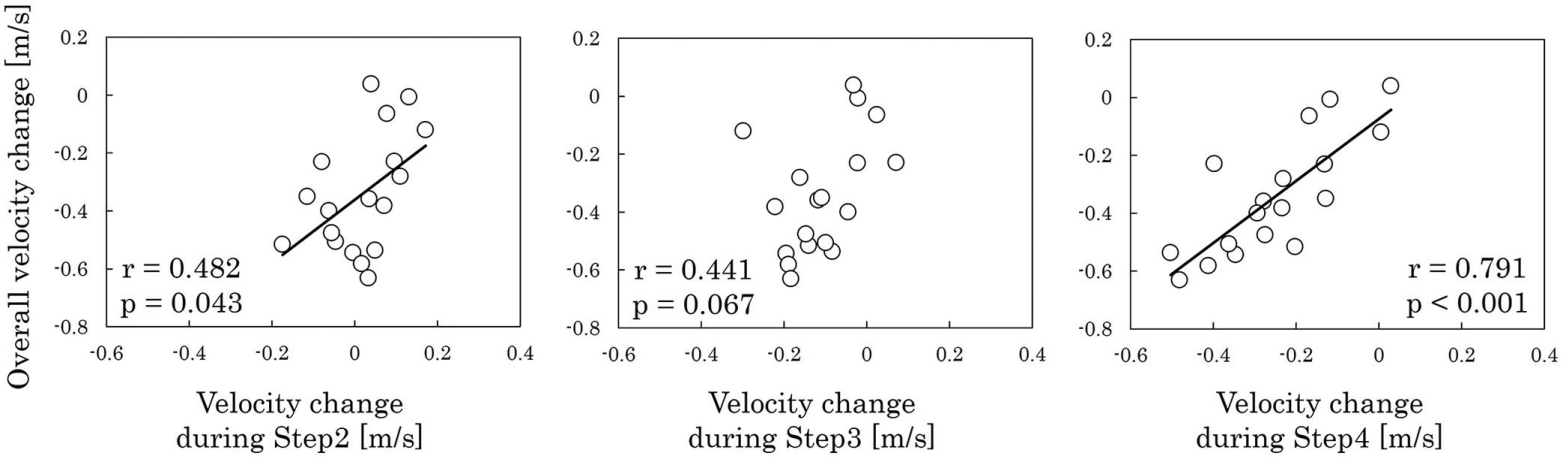
Relationships between overall velocity change throughout the cross-step phase and velocity change at each step.

 Figure 2 presents horizontal CoM velocity from Step1 to Step4. A repeated-measures ANOVA revealed a significant main effect of step ($\eta_{p}^{2}$ = 0.730, *p* < 0.001; large effect). *post-hoc* Bonferroni-adjusted multiple comparisons indicated that Step3 velocity was lower than Step1 and Step2 velocities (Step1 vs. Step3: g = 0.701, *p* = 0.038, medium effect; Step2 vs. Step3: g = 1.112, *p* = 0.002, large effect), and Step4 velocity was lower than Step1, Step2, and Step3 velocities (Step1 vs. Step4: g = 1.603, *p* < 0.001; Step2 vs. Step4: g = 1.916, *p* < 0.001; Step3 vs. Step4: g = 1.585, *p* < 0.001; all large effect). Figure 3 illustrates the relationships between horizontal CoM velocity at R-on3 and both Step1 velocity and overall velocity change throughout the cross-step phase. Horizontal velocity at R-on3 was positively correlated with Step1 velocity (r = 0.890, *p* < 0.001; very large effect), whereas only a small correlation was observed between horizontal velocity at R-on3 and overall velocity change throughout the cross-step phase (r = 0.249, *p* = 0.319; small effect). Figure 4 shows the relationships between overall velocity change throughout the cross-step phase and velocity change at each step. The velocity change during Step2 (r = 0.482, *p* = 0.043; moderate effect) and Step4 (r = 0.791, *p* < 0.001; very large effect) was associated with overall velocity change throughout the cross-step phase.

## 4 Discussion

The primary findings of this study are threefold. First, CoM velocity at the final rear foot contact, which marks the onset of the delivery phase, was strongly correlated with horizontal velocity at the onset of the cross-step phase, whereas it was not strongly associated with overall velocity change throughout this phase. Second, among the four steps, Step3 and Step4 showed lower velocity than preceding steps. Third, velocity change during Step2 and Step4 were associated with overall velocity decrease across the cross-step phase. These findings support our second and third hypotheses, while not supporting the first.

Previous studies have identified CoM velocity at the onset of the delivery phase as an important determinant of javelin throwing performance (8–10, 19). However, velocity dynamics during the run-up and cross-step phases have not been quantitatively examined. To our knowledge, this is the first study to systematically quantify CoM velocity throughout the cross-step phase. The present results indicate that interindividual differences in CoM velocity at the final rear foot contact are more strongly associated with differences in velocity at the onset of the cross-step phase than with differences in velocity change during the cross-step phase. Although velocity at the final rear foot contact can theoretically be expressed as the sum of initial cross-step velocity and subsequent velocity change, only the former showed a strong association with the final velocity in this cohort.

Importantly, the absence of a strong relationship between overall velocity decrease and velocity at delivery onset does not imply that deceleration is mechanically irrelevant. Rather, within this relatively homogeneous group of high-level throwers, variation in initial cross-step velocity was more strongly associated with variation in final approach velocity than variation in velocity decrease during the cross-step phase. In other words, throwers who enter the cross-step phase with higher velocity tend to maintain higher velocity at delivery onset, regardless of the magnitude of subsequent velocity decrease. Although minimizing deceleration during cross-step phase may still be mechanically advantageous, the present findings suggest that, at least among elite throwers, establishing and regulating an appropriate run-up velocity may be more influential than completely preventing velocity decrease during the cross-step phase.

Although CoM velocity remained relatively stable during Step1 and Step2, Step3 and Step4 exhibited more pronounced reduction in velocity. As the final cross step immediately preceding the delivery phase, Step4, the last-cross, requires not only continued forward progression but also preparatory movements for throwing, including extended flight time and trunk positioning. These additional task demands likely increase the mechanical complexity and may render this step more susceptible to deceleration. Additionally, the velocity decrease observed during Step3 may have occurred as an adaptation to the movement demand of the subsequent step.

Furthermore, velocity change during Step 2 and Step4 was associated with overall velocity decrease across the entire cross-step phase, suggesting that deceleration at the last cross substantially contributes to interindividual variability in overall velocity decrease. Previous sprint studies have shown that more anterior foot placement relative to the CoM at ground contact increases braking impulse (20). In running and sprinting, a posterior ground reaction force was observed during the early stance phase (21, 22), when the contact foot is located in front of the CoM. During the cross-step phase, right-handed throwers exhibit a posture in which their torso faces rightward relative to the throwing direction, and this posture becomes more pronounced during the last-cross. This movement may position the left foot more anteriorly at contact, potentially increasing braking impulse. This technical characteristic may partly explain interindividual variability in velocity change during the cross-step phase.

High CoM velocity at front foot contact during delivery phase has been associated with superior throwing performance (3, 23, 24), as has minimizing excessive knee joint flexion after the contact (3). However, achieving a high run-up velocity does not necessarily guarantee improved performance if the thrower lacks sufficient strength or technical capacity to tolerate and effectively transfer the mechanical load imposed at ground contact. A recent guideline on horizontal deceleration ability enhancement states that both strength and technical capacity are required (25). From this perspective, the velocity decreases commonly observed during Step3 and Step4 may not be entirely detrimental. Instead, it may reflect adaptive regulation of run-up velocity to a level compatible with each thrower's physical and technical capabilities. A certain degree of deceleration during the second half of the cross-step may therefore represent a strategy to optimize the balance between approach velocity and effective force application in the subsequent delivery phase. However, these are merely speculations. Future investigation on run-up velocity should consider not only its magnitude but also its optimal regulation for individual throwers.

Finally, several limitations of this study should be acknowledged. First, the analysis was limited to the cross-step phase; therefore, the direct relationship between CoM velocity dynamics during this phase and the subsequent delivery-phase kinematics and throwing performance remains unclear. Future research should examine how velocity characteristics during the second half of the cross-step influence kinematics and release parameters during the delivery. Second, the variability in performance level among participants was relatively small, as all athletes were high-level throwers who had qualified for a national championship. Consequently, the present findings may not be generalizable to athletes with lower or more heterogeneous performance levels. Including a broader range of athletes in future studies may yield different results and provide a more comprehensive understanding of the relationship between cross-step mechanics and performance. Third, this study focused on inter-individual differences. Investigating within-athlete variability in cross-step velocity dynamics would provide further insight into movement consistency and individual regulation strategies. Addressing this issue represents an important direction for future research.

## 5 Conclusion

This study quantified the dynamics of horizontal CoM velocity throughout the cross-step phase in competitive javelin throwing. Horizontal velocity at the final rear foot contact, marking the onset of the delivery phase, was strongly correlated with velocity at the onset of the cross-step phase, but not with overall velocity decrease during this phase. Step3 and Step4 exhibited lower velocities than the preceding steps, and velocity change during Step2 and Step4 was associated with overall velocity decrease across the cross-step phase. Collectively, these findings indicate that, among high-level throwers, interindividual differences in velocity at the onset of the delivery phase are more closely related to initial cross-step velocity than to the magnitude of velocity decrease during the cross-step phase. Nevertheless, because velocity decrease was most pronounced during the second half of the cross step, understanding the mechanical factors underlying deceleration at these steps may provide further insight into cross-step technique and approach velocity regulation.

## Data Availability

The original contributions presented in the study are included in the article/supplementary material, further inquiries can be directed to the corresponding author.
